# The Importance of Bilayer Asymmetry in Biological Membranes: Insights from Model Membranes

**DOI:** 10.3390/membranes15030079

**Published:** 2025-03-03

**Authors:** Igor S. Oliveira, Guilherme X. Pinheiro, Maria Luana B. Sa, Pedro Henrique L. O. Gurgel, Samuel U. Pizzol, Rosangela Itri, Vera B. Henriques, Thais A. Enoki

**Affiliations:** Institute of Physics, University of Sao Paulo, Sao Paulo 05508-090, Brazil

**Keywords:** lipid bilayer, liquid ordered and liquid disordered phases, partition coefficient, asymmetric membranes

## Abstract

This mini-review intends to highlight the importance of bilayer asymmetry. Biological membranes are complex structures that are a physical barrier separating the external environment from the cellular content. This complex bilayer comprises an extensive lipid repertory, suggesting that the different lipid structures might play a role in the membrane. Interestingly, this vast repertory of lipids is asymmetrically distributed between leaflets that form the lipid bilayer. Here, we discuss the properties of the plasma membrane from the perspective of experimental model membranes, consisting of simplified and controlled *in vitro* systems. We summarize some crucial features of the exoplasmic (outer) and cytoplasmic (inner) leaflets observed through investigations using symmetric and asymmetric membranes. Symmetric model membranes for the exoplasmic leaflet have a unique lipid composition that might form a coexistence of phases, namely the liquid disordered and liquid order phases. These phase domains may appear in different sizes and shapes depending on lipid composition and lipid–lipid interactions. In contrast, symmetric model membranes for the cytoplasmic leaflet form a fluid phase. We discuss the outcomes reported in the literature for asymmetric bilayers, which vary according to lipid compositions and, consequently, reflect different intra- and inter-leaflet interactions. Interestingly, the asymmetric bilayer could show induced domains in the inner leaflet, or it could decrease the tendency of the outer leaflet to phase separation. If cells regulate the lipid composition of the plasma membrane, they can adjust the existence and sizes of the domains by tuning the lipid composition.

## 1. Introduction

Biological membranes rely on diverse lipid compositions to function as lipid barriers protecting the cell and its organelles, if present, as well as the regulation of cell function [1]. Eukaryotic cells possess a wide variety of lipids and allocate significant resources to produce hundreds of lipid structures. As van Meer et al. (2008) [2] pointed out, cells utilize 5% of their genetic material to produce a large number of different lipids. A question that arises is the following: Is there a purpose for such a significant expenditure of energy in terms of membrane function?

A relevant property of natural cell membranes is their bilayer asymmetry, which stems from the different lipid compositions of the individual leaflets forming the bilayer. Investigations from the last 50 years have shown that many organisms, including some types of bacteria [3,4,5] and mammalian cells [6,7,8,9,10], present asymmetric lipid bilayers enclosing their cells or organelles. These studies identified the main lipids present in the two leaflets in terms of their headgroups. More recently, Lorent et al. (2020) [11] presented a detailed picture of the phospholipid distribution on each leaflet of the plasma membrane of red blood cells using an enzymatic digestion assay. The authors added information on the distribution of acyl chain length and unsaturation, which play an important role with regard to order in the bilayer. A predominance of nearly saturated phosphatidylcholine (PC) and long chains sphingomyelin (SM) is found in the outer leaflet, while unsaturated phosphatidylcholine, phosphatidylethanolamine (PE), and ionizable phosphatidylserine (PS) compose most of the inner leaflet.

The lipid composition of the biological membrane has implications for its structure and lipid mobility, with the possibility of separating specific lipid domains. The order or fluidity of the lipid structure impacts membrane function in the cell [12]. Phase properties are relevant for protein association and transport across the membrane and have been extensively explored in simple experimental (*in vitro*) or numerical (*in silico*) studies of symmetric bilayers. This has led to the development of a large set of accurate techniques, both for the laboratory and for computers, which allow recognition of the coexistence of phases, such as the liquid ordered, liquid disordered, or gel phases manifested in lipid bilayers. In the last twenty years, these techniques have been used in exploring the asymmetric bilayer.

Knowledge of the main components of the cell membrane’s inner and outer leaflet inspired studies in molecular dynamics simulations, and many numerical experiments have been conducted in the last twenty years, yielding significant advances in the field. For example, Chaisson et al. (2023) [13] carefully review and analyze examples of these simulations.

Experimental investigation of model asymmetric bilayers (*in vitro*) had to wait for the development of innovative techniques [14,15,16,17,18,19,20,21,22,23,24,25,26,27,28,29,30]. Nevertheless, recent advancements in innovative experimental methods have the potential to lead to the emergence of new and significant findings.

In this mini-review, we emphasize important aspects of the experimental models we studied concerning the plasma membrane. First, the diversity of lipids, illustrated here by a few lipid categories, can result in the coexistence of different phases. Second, bilayer asymmetry leads to questions about the membrane phase behavior and the interactions between leaflets of different compositions.

As an initial approach, we concentrate on the phase behavior of lipid mixtures that individually represent either the exoplasmic or the cytoplasmic leaflets, looking for the presence of coexistence properties. This review examines the recent investigations conducted on symmetric membranes, which model these leaflets separately. In the sequence, we compare the results in symmetric bilayers and findings on asymmetric model membranes and discuss their differences.

Simplified biomembranes help to manage different factors more effectively and provide clearer models. These models can explain biophysical principles that support and clarify observations. In Section 2, we delve into the results for the structure of the exoplasmic leaflet, derived from data on symmetric bilayers with the lipid composition of the outer leaflet in the plasma membrane. Section 3 presents the results corresponding to symmetric bilayers with the composition of the inner plasma membrane leaflet. Finally, in Section 4, we explore the results of models for the asymmetric plasma membrane and the question of coupling between the two bilayer leaflets. Section 5 offers some final considerations, highlighting the recent advancements in our understanding of lipid composition and phase behavior.

## 2. Modeling the Exoplasmic Leaflet of the Plasma Membrane

**Figure 1:**
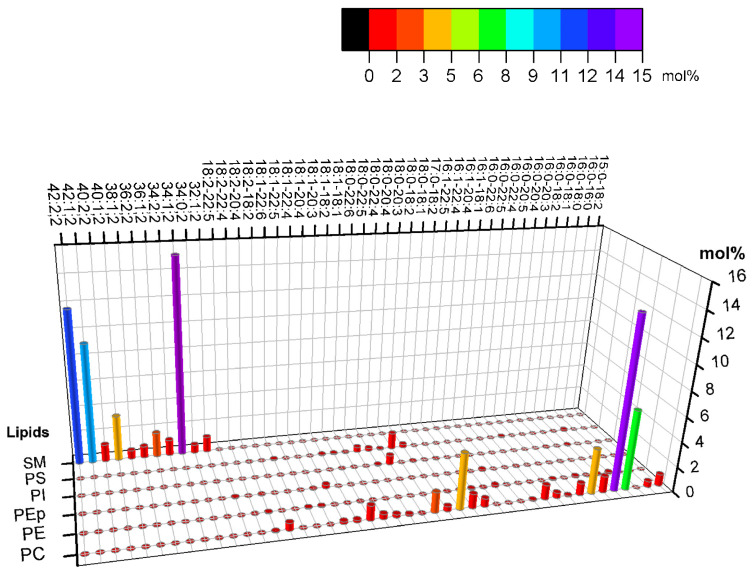
The molar composition of the main phospholipid species of the exoplasmic leaflet of the plasma membrane (PM) is given in mol% according to [11]. The axes indicate the lipid headgroup, number of carbons, level of unsaturation, and lipid molar fraction. A color code highlights the molar fraction for straightforward comparison. For example, PC 14:0–18:1 refers to the phosphatidylcholine headgroup, a saturated acyl chain with 14 carbons, an acyl chain of 18 carbons, and a double bond. The plot shows percentages greater than 1%.

Lorent et al. (2020) obtained the phospholipid constitution of the plasma membrane’s leaflets [11]. Figure 1 shows that the exoplasmic leaflet has a significant fraction of high-melting-temperature lipids (high Tm lipids), such as long-chain sphingomyelin (SM), for which, in general, the main gel–fluid transition temperature is above 40 °C. Figure 1 also shows that the outer leaflet presents a considerable fraction of phosphatidylcholine lipids with much lower melting points due to chain unsaturation. In addition to phospholipids, the plasma membrane also contains cholesterol, a lipid that is important in many cellular processes. The fraction of cholesterol between leaflets is still under debate.

In the following subsections, we present the main results of studies on the lipid structure of the symmetric membrane composed of some phospholipids used to model the outer leaflet of the plasma membrane plus cholesterol (see Figure 1). The phase structure and lipid mobility depend on lipid relative concentrations. Thus, an extensive range of phospholipid relative molar densities can be explored. A very efficient way of illustrating the results of such investigations is to present the results in terms of phase diagrams. Here, we are interested in the phase coexistence of the liquid ordered and disordered phases, which are more relevant from the cell point of view, and in the information on the size of the domains that may reach nanoscales depending on the lipid composition. The presence of nanodomains may provide strong evidence in favor of the lipid raft hypothesis.

In the following, we revise a few phase diagrams relevant to rationalizing the outer leaflet behavior. Here, DSPC (1,2-distearoyl-sn-glycero-3-phosphocholine) or bSM (sphingomyelin (brain, porcine)) stands for the lipids with plasma membranes that have high-temperature melting points; both of these generally have long saturated acyl chains.

### 2.1. Triangular Phase Diagrams

**Figure 2:**
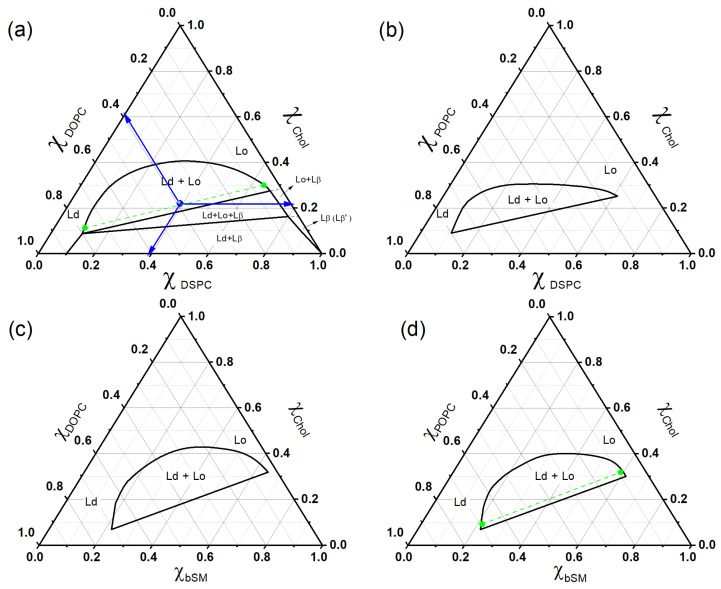
Ternary diagrams for (**a**) DSPC/DOPC/chol, (**b**) DSPC/POPC/chol, (**c**) bSM/DOPC/chol, and (**d**) bSM/POPC/chol, T = 23 °C. In diagram (**a**), the composition coordinates for a particular point P along the axes are indicated as DSPC/DOPC/chol = 0.39/0.39/0.22 (as highlighted by the blue arrows). The lipid mixtures (**a**,**c**) form domains on the scale of micrometers, whereas the lipid mixtures (**b**,**d**) form domains on the scale of nanometers [34,35,36]. The green dashed line indicates the tielines, which are discussed later in the text.

Triangular phase diagrams play a crucial role in deciphering the complex phase behavior of ternary systems in general, and of lipid ternary systems in particular [31,32,33]. These systems involve the physical interaction of three chemical components combined in varied proportions. A typical example of such a system of interest in the study of biomembranes is the mixture of phospholipids and cholesterol [34,35,36], as illustrated in Figure 2.

A triangle represents the full range of relative concentrations of the three-component system. Each vertex represents one of the pure components, while the points on the sides represent binary mixtures. Each point inside the triangle indicates a specific three-component composition.

Different compositions of the ternary system may present different thermal phases at the same temperature. Thus, triangular phase diagrams are valuable for indicating the regions with lipid concentrations that present a single equilibrium phase or regions of coexistence between different phases at a specific temperature.

In Figure 1, we present the phase diagrams for (a) DSPC/DOPC/chol, (b) DSPC/POPC/chol, (c) bSM/DOPC/chol, and (d) bSM/POPC/chol. Panel (a) illustrates various phases and regions of coexistence. We also provide an example of how to interpret a specific lipid composition: in this example, DSPC/DOPC/chol is represented as 0.39/0.39/0.22. The subsequent diagrams concentrate on the two-phase region, which is crucial for the discussion in this mini-review article.

Determining tielines can be challenging. Previous studies have explored the use of wide-angle X-ray scattering (WAXS) on oriented lipid multilayers of DPPC/DOPC/chol to establish the tielines of this ternary mixture [37]. Additionally, a different method employs electrofusion vesicles to determine the tielines of eSM/DOPC/chol [38]. These studies demonstrate that not all tielines are parallel throughout the entire two-phase region. However, for low-cholesterol fractions, the tielines tend to run parallel to the line that separates the three-phase region from the two-phase region.

### 2.2. Mapping the Coexistence Region Ld + Lo Phases

Different experimental techniques may be used to investigate the coexistence of phases. The most straightforward method is fluorescence optical microscopy, which uses fluorescent labels that prefer one specific phase. In addition to microscopy techniques, one may investigate the presence of phase coexistence using different techniques, such as steady-state probe partition (SSPP) fluorescence [31,34,35,36,39,40,41,42], electron spin resonance (ESR) [40,43], small-angle X-ray scattering (SAXS) [44], small-angle neutron scattering (SANS) [35,45], cryo-EM [46,47], and atomic force microscopy (AFM) [48,49]. Here, we present data that are mainly based on fluorescence techniques.

Fluorescence techniques rely on information given by the fluorescence of dye molecules. In the presence of phase coexistence, dye molecules in the membrane may prefer one phase over the other, yielding a partition of such molecules. The experimental techniques based on fluorescence use the concept of partition to investigate the properties of different phases.

The partition coefficient (*K_P_*) represents a measure of the preference of a specific molecule for one phase over another in terms of the ratio of the dye fractions in each of the two phases in equilibrium, as shown in Equation (1). It is usual to define a partition coefficient greater than 1 to indicate a higher preference for the disordered phase. In contrast, a partition coefficient lower than 1 indicates a higher preference for the ordered phase (e.g., ([36])).

(1) $$K_{P}=\frac{\left(f_{Ld}/\chi_{Ld}\right)}{\left(f_{Lo}/\chi_{Lo}\right)}$$

where $f_{Ld}$ and $f_{Lo}$ are the fractions of fluorescent probes that partition into the Ld and the Lo phases, and $\chi_{Ld}$ and $\chi_{Lo}$ are the fractions of these phases, respectively.

We can estimate the partition coefficient for fluorescent dyes from confocal microscope images. Alternatively, we can extract the *K_P_* from the steady-state probe partition (SSPP) experiment—fluorescence or SSPP-FRET, as discussed below [50].

#### 2.2.1. Fluorescence Microscopy

**Figure 3:**
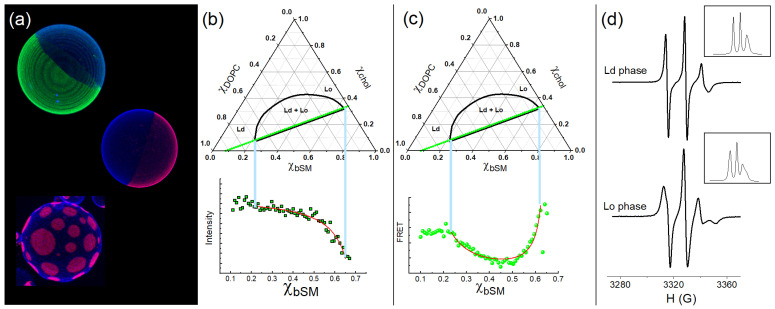
Examples of different techniques to study the coexistence of Ld + Lo phases. (**a**) Images of GUVs obtained on a confocal microscope exhibiting the coexistence of phases Ld + Lo. The Ld phase is colored green and pink, and the Lo phase is blue. (**b**) The graphs illustrate the phase diagram of bSM/DOPC/chol (top panel) and the fluorescence intensity of Bodipy-PC (bottom panel), a fluorescent probe incorporated into the trajectory of samples indicated in the phase diagram. The red line represents the adjustment of equation 2. (**c**) The graphs illustrate the phase diagram of bSM/DOPC/chol (top panel) and the FRET signal between DHE and Bodipy-PC (bottom panel). The donor probe, DHE, is partitioned into the Lo phase, whereas Bodipy-PC prefers the Ld phase. The red line represents the fitting of equation 3. (**d**) The graphs show the ESR signals typical of the Ld (top panel) and the Lo (bottom panel) phases for the lipid mixture DSPC/DOPC/chol. Inset graphs show the absorption spectra. Data adapted from [36,40]. Experiments performed at T = 23 °C.

Figure 3a displays examples of giant vesicles labeled with different fluorescent probes. The authors captured these images using confocal microscopy [36,40]. The fluorophores indicate the coexistence of two phases, namely the Ld and Lo phases. In this example, the Ld phase is labeled green or pink.

The phase diagrams in Figure 2 for the DSPC/DOPC/chol and bSM/DOPC/chol compositions correspond to samples that display visible domains under optical fluorescence microscopy [35,36,39,51,52]. Note that DSPC and bSM are high Tm lipids, whereas DOPC is a low Tm lipid due to unsaturation on the acyl chains.

Interestingly, when POPC replaces DOPC, where POPC has one of the unsaturated acyl chains, the samples of DSPC/POPC/chol and bSM/POPC/chol, which are expected to show Ld + Lo domains, form giant vesicles with a uniform appearance [53,54]. Could there be phase domains whose sizes fall below the threshold of optical resolution? The study of various experimental methods was necessary to evaluate the presence of submicron domains, potentially on a nanoscale scale. Next, we briefly describe a few techniques that can be employed to investigate the coexistence of phases for nanoscale domains.

#### 2.2.2. Steady-State Probe Partition Fluorescence

Fluorescent probes might exhibit different fluorescence intensities when embedded in distinct environments [55]. This observation can relate to different fluorescence quantum yields in each phase. As fluorescent probes partition between different environments, we can calculate the partition coefficient based on the amount of dye in each phase, which is measured by the fluorescent signal in a trajectory of samples. In these experiments, the samples have slightly different lipid compositions (e.g., the fraction of bSM is increasing by 0.01), as illustrated in the phase diagram by green dots. Figure 3b shows the fluorescent signal of each composition. To be noted is the brighter signal in the Ld phase compared to the Lo phase. Thus, the fluorescent signals from the samples in the two-phase region can be described as a combination of signals from the samples at the timeline endpoints, namely the compositions of a pure Ld phase on the tieline left-hand side and a pure Lo phase on the right-hand side [36]. The fluorescence intensity for the samples belonging to a tieline can be described by Equation (2):

(2) $$I=\frac{\chi_{Lo}I_{Lo}+K_{P}(1-\chi_{Lo})I_{Ld}}{K_{P}(1-\chi_{Lo})+\chi_{Lo}},$$

where $K_{P}\;$ is the partition coefficient, as described by Equation (1), and $I_{Ld}$ and $I_{Lo}$ are the intensities measured on the tieline endpoints.

The steady-state probe partition fluorescence can measure the partition coefficient of the fluorescent molecules that partition between the Ld and the Lo phases. However, based solely on this analysis, we cannot determine the domains’ sizes for the different phases.

#### 2.2.3. Steady-State Probe Partition—Förster Resonance Energy Transfer

Förster resonance energy transfer (FRET) employs the study of energy transfer between two dye molecules instead of monitoring the fluorescence signal of a single dye particle. This technique makes it possible to estimate distances on the nanometer scale. The energy transfer between fluorescent donors and fluorescent acceptors depends on the distance between both fluorophores. The FRET efficiency increases as dye molecules get closer and decreases when the donor and acceptor molecules are far apart. The distance between two fluorescent probes is affected by each fluorophore’s preference for a specific phase [36,40].

Figure 3c shows an example where different probes, DHE and Bodipy-PC, prefer different phases. For this case, the region in the composition space of the phase diagram with a lower FRET efficiency indicates a region with a coexistence of phases. DHE prefers the Lo phases, whereas Bodipy-PC prefers the Ld phase. It can be seen that FRET efficiency (or the monitored FRET signal) varies abruptly at the phase coexistence endpoints at which the FRET pair (donor and acceptor probes) partition into their preferred phase. The FRET intensity for the samples belonging to a tieline can be described by Equation (3):

(3) $$FRET=\frac{F_{Ld}K_{P}^{A}K_{P}^{D}(1-\chi_{Lo})+F_{Lo}\chi_{Lo}}{\left[K_{P}^{A}+(1-K_{P}^{A})\chi_{Lo}\right]\;[K_{P}^{D}+(1-K_{P}^{D})\chi_{Lo}]},$$

where $K_{P}^{D}$ and $K_{P}^{A}$ are the partition coefficients of the donor and the acceptor probes, and $F_{Ld}$ and $F_{Lo}$ are the FRET signal measured on the tieline endpoints.

#### 2.2.4. Electron Spin Resonance to Monitor Lipid Packing/Order

Other techniques are advantageous in investigating the coexistence of the Ld and the Lo phases. Electron spin resonance (ESR) can offer supplementary evidence regarding domains exhibiting distinct physical properties, such as the lipid packing/order. In ESR experiments, we employ a paramagnet probe with a spin quantum number of S = 1/2. In the presence of an external magnetic field, H(G, Gauss), the electron’s magnetic moment aligns itself either antiparallel or parallel to the field; this is known as the Zeeman effect. In addition, the hyperfine structure causes the splitting of the electronic energy levels. The radiation absorption in the microwave frequency range leads to the spectra shown in the insert of Figure 3d. Figure 3d shows the first derivative of the ESR absorption spectrum (ESR signals) obtained for the Ld (upper panel) and the Lo (lower panel) phases of DSPC/DOPC/chol, as previously reported [40]. The line width and the height of the peaks are related to the motion of the paramagnetic probe. The phenomenological parameters emanating from the ESR signal can also describe lipid packing/order. Alternatively, one can simulate the ESR signal following a defined methodology, which involves solving the spin Hamiltonian and finding parameters that adjust to the experimental data [56]. In this mini-review, we refrain from providing a formal definition of order, as we are not using any parameter to compare lipid packing. Therefore, we utilize the term “lipid packing/order” to convey our concepts effectively.

#### 2.2.5. Pros and Cons of These Techniques

Microscopy fluorescence techniques can immediately identify the coexistence of phases when the sizes of the lipid domains are above approximately 200 nm. Utilizing this technique involves creating GUVs; the most predominant method, known as Electroformation, typically produces a compositional error of roughly 5 mol%. In addition, GUVs with a lipid mixture of three or four lipids are prepared at a temperature above the main transition of those lipids to ensure ideal mixing. The cooling rate of these GUVs to room temperature could also affect domain sizes, as reported [42,52]. Thus, this technique may not be the most accurate to map phase diagrams.

On the other hand, by meticulously preparing a series of samples, as depicted in Figure 3b,c, and adhering to strict protocols for controlling lipid concentration and fractions, we can significantly minimize the composition error to just 3 mol% (see, e.g., [36]). These fluorescence and probe partitioning techniques use vesicles prepared by rapid solvent exchange, and the procedure yields vesicles with one or two lamellas and sizes of a few hundred nanometers. One of the most compelling benefits of these techniques is their ability to generate various samples. For example, the analyses in Figure 3b,c show the fluorescence or FRET signals measured from 61 samples.

Nevertheless, SSPP fluorescence is ineffective for delineating the two-phase region’s phase boundaries. For instance, the phase boundary on the left side (between the Ld and Ld + Lo regions) does not appear distinctly in the SSPP fluorescence signal. This plateau in the fluorescence signal is associated with a high Kp, which favors the Ld phase.

Interestingly, SSPP FRET is very efficient in demarcating the two-phase region, especially when experiments are planned with a FRET pair that partitions into different phases, as in the case of DHE and Bodipy-PC. As illustrated in Figure 3d, the FRET signal from the samples in the two-phase region differs from the signals observed in a single-phase region. In addition, this technique is not limited to domain sizes constrained by optical resolution, highlighting a key advantage that sets this method apart from others. Using FRET, as the energy transfer relies on the separation distance between donors and acceptors, we can evaluate the signal from the separation distances on a nanometer scale. In other words, the energy transfer occurs in a range of up to 2 R_0_, where R_0_ is the Forster distance, which has dimensions of a few nanometers. R_0_ depends on the FRET pair; in the example of Figure 3d, R_0_ = 2.8 nm. Thus, it is possible to distinguish the FRET signal when donors and acceptors are (i) randomly distributed in a single phase, (ii) far apart in large domains (the signal from the domain boundary is negligible), and (iii) far apart in small domains (the signal from the domain boundary is not negligible).

The Ld and Lo phases display different properties, such as lipid packing/order, bilayer thickness, and bending moduli. Experiments using ESR are valuable for identifying differences in lipid packing and order, as the paramagnetic signal reveals distinct variations in more or less packed environments. However, despite the advantages of characterizing the properties of each phase, it is important to note that this method may not be well suited for marking phase boundaries. The ESR signal of the coexistence phase results from combining signals from the individual phases, making the analyses straightforward when the phase diagram and tielines are known.

### 2.3. Alignment of Domains Within a Bilayer

The literature often delves into the complex topic of the alignment of lipid domains across the bilayer in symmetric or asymmetric bilayers, in which both leaflets have lipid composition-forming domains. This discussion focuses on how these domains self-organize across the bilayer and whether they maintain a coordinated orientation between the two leaflets of the membrane. For that, we use the jargon of “domain registration.” For example, the non-alignment of the Lo domains (assuming infinitely rigid domains) suppresses the membrane undulations. Thus, the stiff domains in individual leaflets are driven to be in registration, allowing membrane undulations and, therefore, maximizing the entropy [57]. Another possible explanation, according to a theoretical model proposed by [58], is that the line tension may be enough to facilitate domain registration, leading to a small area of phase mismatches at the domain boundary [58].

This mini-review does not focus on this discussion. In the later sections, we discuss asymmetric bilayers and highlight the difference in phase behavior of each leaflet that forms the plasma membrane’s mimetic bilayer. A key aspect of this model is that one leaflet forms a coexistence of phases, whereas the other leaflet forms a single fluid phase.

### 2.4. Quaternary Phase Diagrams

Investigation of ternary lipid mixtures has shown that a lipid mixture, such as DSPC/DOPC/chol, forms macrodomains that we can observe under the microscope, whereas a different lipid mixture, such as DSPC/POPC/chol, forms phase domains of a nanometer scale, as observed with various spectroscopic techniques. As different lipid compositions yield domain sizes that might differ by several orders of magnitude, an interesting question arises: What happens when we mix these four lipids DSPC/DOPC/POPC/chol? What kinds of domains will we observe?

Feigenson’s laboratory developed an answer to this question. The group studied different four-component mixtures, including DSPC/DOPC/POPC/chol, to investigate the morphology of the phase domains under relative fraction variations [39,53]. The ratio between DOPC and POPC was varied, while the cholesterol and DSPC percentages remained fixed (χchol = 0.22 and χDSPC = 0.39). The authors defined a density variable, as shown in Equation (4), with 0 < ρ < 1:

(4) $$\rho \;\equiv \;\frac{\chi DOPC}{\chi DOPC+\chi POPC}.\;\;\;\;\;\;\;\;\;\;$$

Experiments performed at room temperature yielded the formation of different morphologies of the Ld/Lo domains for different ranges of the relative fraction ρ.

**Figure 4:**
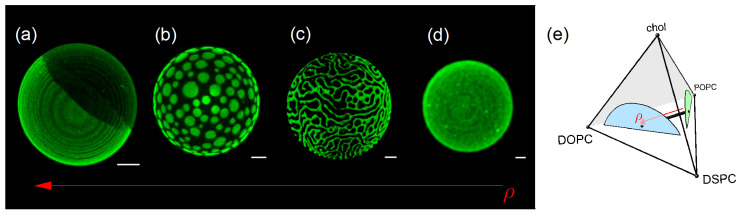
Images of a DSPC/DOPC/POPC/chol GUV were obtained using a confocal microscope; data were adapted from [59]. (**a**–**d**) The fraction of DOPC increases from right to left, while the fraction of POPC decreases. See the text for the definition of ρ, Equation (4). The green color indicates Ld, while black indicates Lo. (**a**) The image shows a complete phase separation. (**b**) The image shows domains of the Ld phase in the continuous Lo phase. (**c**) The image shows an example of modulated phases. The quaternary phase diagram is illustrated in (**e**). The arrow in the phase diagram illustrates the lipid compositions of the GUVs.

Figure 4 illustrates the microscopy results with different values of ρ. For ρ = 1 (Figure 4a), we observe complete phase separation of the Lo (black) and Ld (green) phases. For ρ = 0.45 (Figure 4b), we can see many circular Ld domains in a background “sea” of the Lo phase. For ρ = 0.32 (Figure 4c), the domains form different patterns, with either a honeycomb appearance or strips, called modulated phases. Finally, for ρ = 0 (Figure 4d), giant vesicles show a uniform appearance under the microscope. However, as mentioned above, domains have sizes of a few nanometers and cannot be observed under the microscope.

### 2.5. Energetic Factors Driving Phase Separation and Competing Interactions

As mentioned, various simplified lipid mixtures of three or four lipid components display a liquid-ordered and liquid-disordered coexistence at a specific temperature [35,36,39,40,53,60,61,62,63]. For the examples forming macrodomains, such as those for ρ = 1, the phase separation minimizes the interface line between the two phases, which is a consequence of minimizing the system’s free energy. At coexistence, the system’s free energy includes contributions from the free energies of each bulk phase and from the interface. The interface free energy is proportional to the interface’s length, with the interface tension as a linear coefficient. The minimum free energy corresponds to the minimal length of the interface line. The line tension is reported to influence domain size and morphologies [40,64]. We can measure the domain line tension using flicker spectroscopy [65].

Modulated phases, however, have been seen for many lipid compositions (e.g., [40]). The presence of modulated phases implies a longer interface line, given by the sum of the domain interface lines. Thus, there should be some compensation for the penalty for a larger interface.

Modulated phases with length scales ranging from centimeters to nanometers are exhibited by very different physical systems, such as colloidal or magnetic systems, as has been reviewed by [66]. The authors show that competing interactions, such as an additional repulsive interaction added to the attractive interaction, which favors phase separation, may yield modulated phases [66]. If only attractive interactions are present, the system may present either complete phase separation (at a lower temperature) or random mixing (at a higher temperature) due to energy–entropy competition. The repulsive interaction raises the bulk free energy, which incorporates energy and entropy effects and thus favors the breaking-up of the two-phase domains, producing competition with the interface free energy. Under the thermodynamic conditions in which the latter wins, the system will be unstable in terms of the formation of domains. Otherwise, if the repulsive interaction wins, the competition of interactions may yield smaller domains of one phase embedded in the second phase, depending on temperature and other thermodynamic parameters.

Numerical simulations were used to investigate the effect of surface elastic deformation [67] or a repulsive force between headgroup dipoles [68] on the domain structure of coexisting phases.

Both effects, which compete with the line tension between the Ld and the Lo phases, can reproduce fragmentation of the phases, depending on the model parameters. Numerical experiments used a discretized model of a spherical fluctuating surface (2013), whose energy was described by the Helfrich Hamiltonian [69], which presents a positive contribution from local curvature and a negative contribution from local Gaussian curvatures. The authors adjusted the model parameters to produce modulated phases. The parameters thus obtained are about ten times smaller than the experimentally measured value for line tension, whereas the experimental data’s elastic constants are within the same range.

In a second paper (2014), the authors probe the role of dipole–dipole interactions between the zwitterionic headgroups and the importance of surface curvature in the appearance of nanodomains or modulated phases [68]. The authors use the radial distribution function to establish the different morphologies. On the other hand, by introducing dipole interactions, the repulsion between lipids favors Lo–Ld contacts. Thus, it competes with line tension, which favors Lo–Lo or Ld–Ld contacts. Consequently, dipole repulsion induces the formation of smaller domains. Additionally, the addition of curvature energy may lead to the emergence of modulated phases.

### 2.6. Membrane Phase Domains and Protein Partition

When two phases coexist, a direct implication is the molecular partition between these phases [70,71,72]. As mentioned above, lipids display a partition described by a thermodynamic tieline. Experimentally, the lipid partition is obtained from the partition coefficients of molecular dyes since different fluorescent molecules move to the corresponding preferred environments, as we saw in the previous sections. When we introduce peptides, proteins, or any external molecules into lipid membranes containing different phases, they may distribute into a specific phase due to physical interactions with lipids. Interestingly, in biomembranes, protein–lipid interactions could also drive transmembrane proteins to a particular phase domain.

The presence of specific environmental conditions can render proteins functional [73]. The fluorescence assay methods mentioned above, which allow the measurement of any labeled molecule’s partition coefficient, are especially pertinent when investigating the partition of peptides and proteins. Thus, studies of model membranes incorporating synthetic peptides can improve the representation of cellular plasma membranes, especially regarding transmembrane proteins.

#### 2.6.1. Transmembrane Peptide Mismatch

Synthetic peptides are used to represent the interactions between the proteins embedded in the membrane and the lipids that compose the lipid bilayer in a simplified manner. Thus, one may investigate the mismatch problem between protein length and membrane thickness using synthetic peptides. The disparity in thickness vs. length occurs when there is a greater chance that the hydrophobic section of the membrane bilayer or the peptide is more exposed to water because of a difference between the bilayer thickness and the peptide length [74].

**Figure 5:**
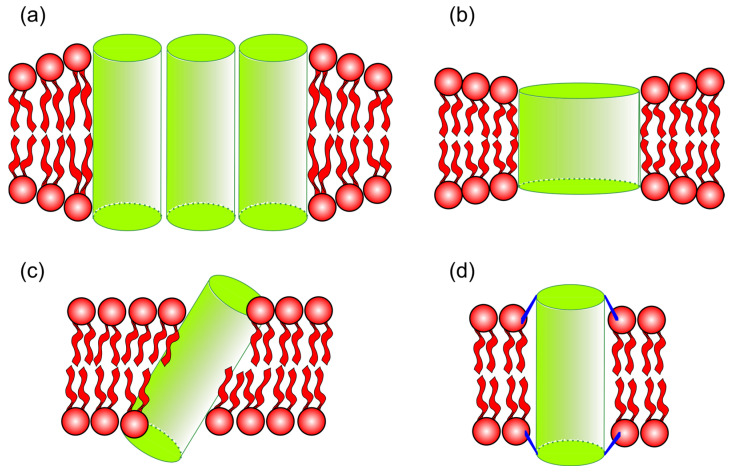
Thickness mismatch between proteins and the lipid membrane. The cylinders represent transmembrane proteins. (**a**,**b**) The cartoons represent positive and negative thickness mismatches. (**c**,**d**) The illustration represents adjustments of the protein to diminish the thickness mismatch. (**c**) The protein is oriented at an angle relative to the normal of the bilayer. (**d**) The protein can adjust lateral residues.

This discrepancy results in an energetically unfavorable interaction, prompting responses from both the peptide and the lipids in contact with the peptide to minimize the mismatch, as shown in Figure 5. There are two types of mismatches: positive and negative. Positive mismatch occurs when the peptide is larger than the thickness of the bilayer, while negative mismatch occurs when the peptide is smaller than the thickness of the bilayer. Among the responses that peptides can exhibit are the following: (i) the formation of peptide oligomers, where peptides aggregate into “bundles” to reduce the contact surface of the peptide’s hydrophobic part with the polar region; (ii) changes in their molecular structure, where peptides larger than the bilayer could, for example, reorganize into a π-helix structure (positive mismatch), and smaller peptides could reconfigure their molecular structure into a 3_10_-helix; (iii) tilting in the direction normal to the bilayer, thus reducing their effective size within the bilayer and protecting the peptide’s apolar region (occurring only for positive mismatch), as previously reported [74,75]; and (iv) reorientation of side chains, flanking residues, near the lipid/water interface, resulting in a reduction in the hydrophobic region’s size.

Notably, the vesicle seeks to optimize energy balance. Among all adaptations, aggregation and reorientation of the side chains stand out as the most efficient responses to mismatch [74]. Additionally, peptide flanking residues are crucial in adjusting the mismatch, with different interactions for positively charged and aromatic residues.

#### 2.6.2. Protein Partition

Regarding the partition of proteins into Ld and Lo phases, Frewein et al. (2016) [76] reported a mathematical model that considers the energetic contributions to protein partitioning based on the protein’s size, shape, and overall oligomerization state. This model uses experimental values for the Lo/Ld domains, such as thickness, intrinsic curvature, bending rigidity, and Gaussian curvature modulus.

**Figure 6:**
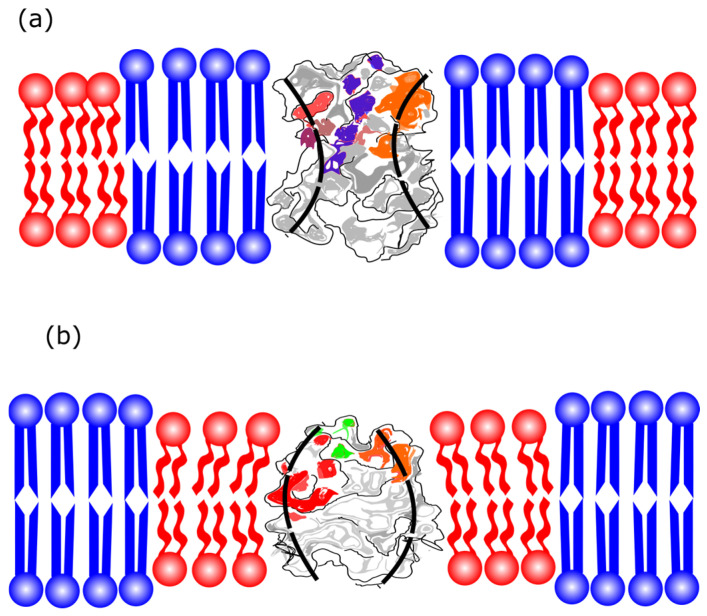
The images illustrate the phase preferences of protein localization in symmetric bilayers according to their shape. The cartoons were inspired by the model proposed by [76]. (**a**) A concave protein prefers ordered domains. (**b**) A protein with a convex shape favors disordered domains.

The findings show that proteins with a concave shape tend to cluster more in the Lo domains, Figure 6a, while proteins with a convex shape have a greater tendency to locate in the Lo domains, Figure 6b. This preference becomes more pronounced as the proteins increase in size [76].

A range of experimental methodologies is available for incorporating proteins and peptides into lipid bilayers. Many studies focusing on the transmembrane peptides of the WALP family involve preparing symmetric bilayers with the peptides already embedded within the lipids [77,78,79,80,81]. Additionally, Efolodi et al. (2024) proposed a method for adding proteins by transferring them from supported lipid bilayers (SLBs) to giant unilamellar vesicles (GUVs) [82], using a technique similar to the hemifusion method [18], the first method to describe the hemifusion between them [18]. It is important to note that introducing proteins from an external aqueous solution, when possible, may lead to different mechanisms of interactions with the lipid bilayers [83].

Additionally, giant plasma membrane vesicles (GPMVs) are a valuable tool for studying protein partition. These large spherical vesicles are formed from the plasma membrane and separated from various cell types after a specific chemical treatment [84]. As components of the plasma membrane, GPMVs provide a representative sample of the lipids and proteins present within it. Notably, Lorent et al. (2017) present a comprehensive study on the determinant factors of protein transmembrane domains, which influence their partition into ordered phases [71].

## 3. The Cytoplasmic Leaflet of the Plasma Membrane

**Figure 7:**
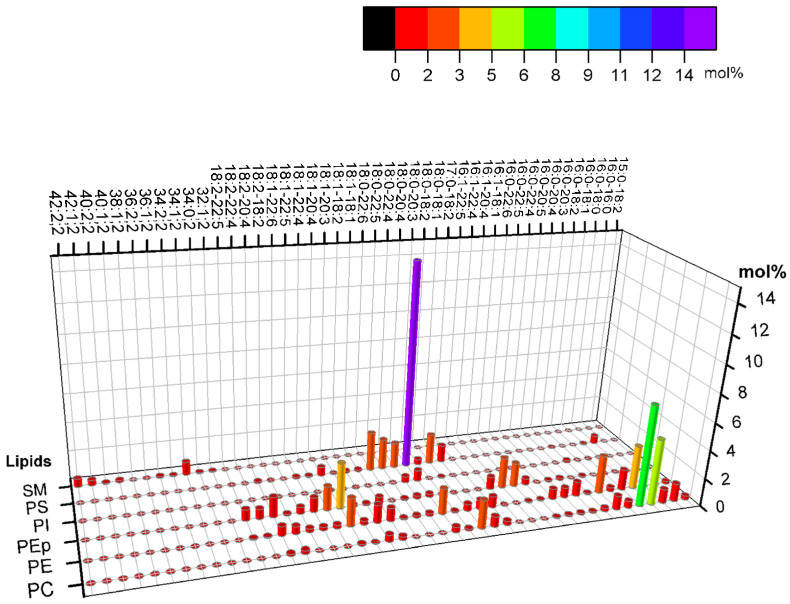
The molar composition of the main phospholipid species of the cytoplasmic plasma membrane leaflet (PM) is given in mol%, according to a previous report [11]. The axes indicate the lipid headgroup, number of carbons, level of unsaturation, and lipid molar fraction. A color code highlights the molar fraction for straightforward comparison, as described in Figure 1.

The lipid composition of the cytoplasmic leaflet of the plasma membrane includes a significant fraction of PC, PE, PEp (PE plasmalogen), and PS head lipids. The asymmetry of PE and PS lipids may be crucial for differentiating between healthy and unhealthy cells since these lipids are predominantly found in the inner leaflets of healthy cells. Conversely, cells that are signaling for death might expose PS, for instance [85]. Additionally, the absence of high Tm lipids and the abundant fraction of polyunsaturated lipids are also the main differences between the cytoplasmic and the exoplasmic leaflets. The average level of unsaturation per lipid in the cytoplasmic leaflet is approximately twice as high as that of the exoplasmic leaflet, underscoring the importance of this composition and the significant difference in unsaturation levels. According to Lorent et al. [11], Figure 7 shows the variety of lipids in the plasma membrane’s cytoplasmic leaflet.

In addition, Wang and Silvius (2001) [86] have investigated symmetric bilayers that mimic the cytoplasmic leaflet composition before the detailed knowledge of the acyl chain properties became available [11]. Inspired by the information that the cytosolic leaflet of the cell plasma membrane presented ordered rafts, the authors investigated the possibility of forming nanoscale domains, in lipid mixtures with PS, PE, and PC phospholipids plus cholesterol. None of these combinations composing the symmetric vesicles displayed ordered domains.

Studies of symmetric bilayers with the composition of the outer (or the inner) leaflet of the plasma membrane on both leaflets reveal that the phase behavior of the two distinct symmetric bilayers is different. According to investigations of symmetric vesicles, those with the composition of the exoplasmic leaflet should present phase separation, while those with the composition of the cytoplasmic leaflet would present a single fluid phase, highlighting the complexity and unique characteristics of each leaflet of the plasma membrane, if treated “independently”.

The study by Wang and Silvius [86] raised a significant question that experimentalists have since pursued: How can these different leaflets interact with each other? This question serves as a crucial starting point for further exploration and understanding of the behavior of asymmetric bilayers.

One of the answers could be an interaction between the two different leaflets, a hypothesis that prompted inquiries into the possibility of mutual influence between the leaflets that compose an asymmetric bilayer.

## 4. The Asymmetric Membrane

Thus far, we have outlined several significant facets of symmetric vesicles with compositions reminiscent of the outer and inner leaflets of the plasma membrane. In this section, we address the bilayer asymmetry and the differences in behavior that arise when the PM exoplasmic and cytoplasmic leaflets compose a model bilayer from experiments on *in vitro* asymmetrical membranes.

Comparing Figure 1 and Figure 7, the composition differences between the exoplasmic and cytoplasmic leaflets are notable. The exoplasmic leaflet has a sizeable fraction of lipids, which form ordered phases. Regarding the inner leaflet, the lipids predominantly yield disordered phases at ambient temperatures. The study of symmetric lipid bilayers alone is limited in its ability to fully capture the complexity of natural cell membranes. Thus, better models of biological membranes must include their asymmetry. Experimental models of asymmetric bilayers could provide valuable insights into the cell membrane structure and function.

In part, the investigation of asymmetric membranes was delayed because preparing asymmetric bilayers in experimental models was difficult. However, the last 10–20 years saw advances in the development of cutting-edge techniques to manage asymmetrical lipid bilayers. In the following sections, we explore some of these methods.

### 4.1. Experimental Procedures to Make Asymmetric Lipid Bilayer

Different methods for preparing and studying asymmetric lipid bilayers have emerged in the last two decades. Krompers and Heerklotz (2023) [87] and Scott et al. (2021) [88] have summarized detailed studies of the literature on these novel methods.

One approach involves creating bilayer asymmetry by modifying the headgroup of some lipids on the outer leaflet with the help of specific enzymes [89,90]. An example of such an enzyme is phospholipase D (PLD), which can convert the PC headgroups into PS (or PE) headgroups via transphosphatidylation in the case that serine (or ethanolamine) is present in the solution and acts on the PC lipids on the outer leaflet. Thus, one may reproduce a feature of natural plasma membranes where the PS and PE headgroups are more numerous in one of the leaflets. This method investigates the bilayer asymmetry regarding the role of lipid headgroups.

**Figure 8:**
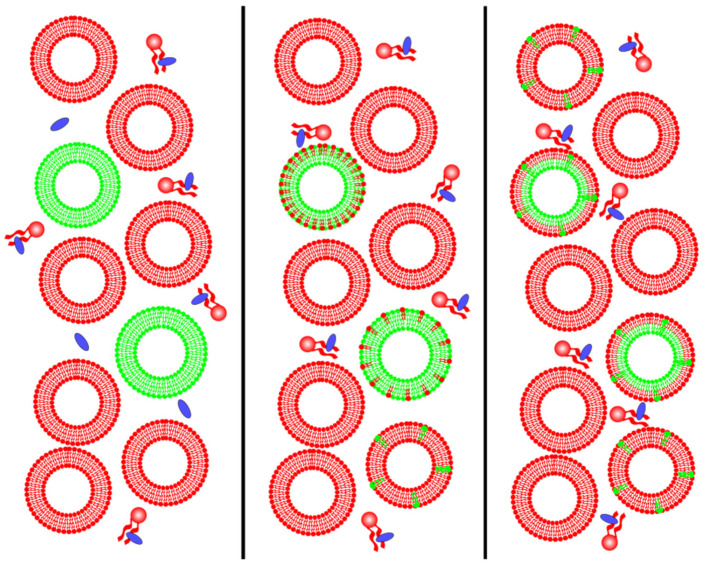
To prepare asymmetric vesicles by the action of cyclodextrins (CDs), represented in blue, CDs are put in a solution in which there are few vesicles with the lipid composition wanted for the inner leaflet (represented in green), and many vesicles with the lipid composition wanted for the outer leaflet (represented in red). CDs carry lipids from the red to the green vesicles and vice versa, forming asymmetric vesicles.

A different approach that has led to significant findings involves cyclodextrins (CDs), which are water-soluble cyclic oligosaccharides displaying amphiphilic properties. With a ring shape whose core is hydrophobic and whose outer side is hydrophilic, CD can involve and carry other molecules. Cyclodextrins come in various sizes, allowing them to interact with various lipids. One or two CDs can load and transport cholesterol if their ring is large enough to fit cholesterol inside, whereas smaller rings can more easily load fatty acids. To create asymmetric vesicles, we can place CDs in a solution with a small number of symmetric vesicles with the desired molecular composition for the inner leaflets and a large number of symmetric vesicles with the desired molecular composition for the outer leaflets (see Figure 8 below). Cyclodextrins molecules randomly exchange the lipids on the outer leaflet between different vesicles, thus forming asymmetric bilayers [23,24,25,26,91,92,93,94,95].

In previous work using CDs as mediators of lipid exchange, the authors reported calculating the lipid exchange percentage using an average value due to the challenge of monitoring individual vesicles. In this procedure, employing MβCD to prepare asymmetric GUVs and achieve a selective exchange of phospholipids is advisable [26].

**Figure 9:**
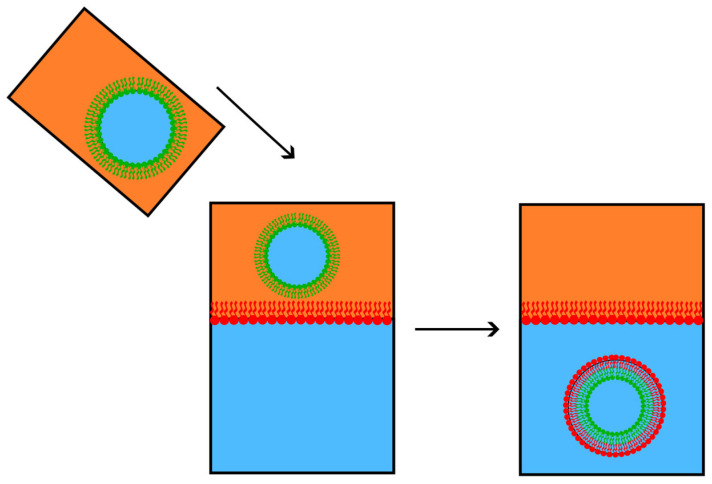
The sequence represents the water–oil system technique for preparing asymmetric membranes. Oil (orange) is used as an apolar medium to prepare inverse micelles with one type of lipid (green). The oil–lipid solution is added to water (blue) without mixing. A second kind of lipid (in red) is added to the oil phase and arranges itself as a monolayer, with the polar heads turned to the water phase. The inverse (green) micelle in the oil phase precipitates through the monolayer into water. It gains an outer (red) monolayer, thus forming an asymmetric vesicle composed of red and green monolayers.

A different strategy for mounting asymmetric bilayers relies on inserting lipids into a water–oil biphasic system, Figure 9. Lipids placed into the oil with water droplets form a reverse micelle, in which the hydrophobic chains turn to the oil phase; these are the lipids that resemble the composition of the inner layer of the asymmetric membrane. In addition, another mixture of lipids, chosen to represent the composition of the outer leaflet, is dissolved in a separate container at the interface of a two-phase water–oil system. The next step is to put the first solution with the reverse micelles into this container. As water droplets fall from the oil to the water phase, the lipids of the inverted micelles interact with those of the oil–water interface and coat the micelles, forming an asymmetric bilayer vesicle in the water [96], Figure 9. However, an undesired effect is that oil residues remain between leaflets. Moreover, recent reports pointed out the asymmetry might be less than 100%, as initially expected [97]. Nevertheless, a few studies report the percentage of asymmetry or possible contamination of lipids expected to be in the inner leaflet and to stay in the outer leaflet and vice versa.

**Figure 10:**
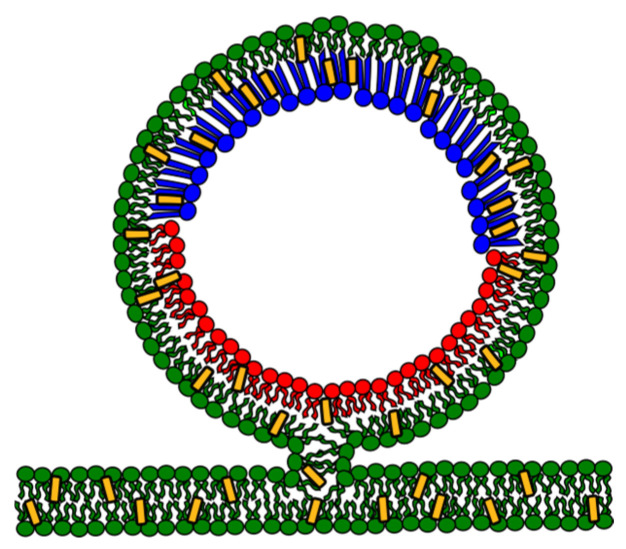
Illustration of the hemifusion between a giant unilamellar vesicle (GUV) and a supported lipid bilayer (SLB). Lipids of different colors represent an Ld (red) and an Lo (blue) phase, respectively. The SLB comprises an Ld phase (dark green) of a distinct lipid composition. Cholesterol is represented in yellow. During hemifusion, the outer leaflets of the GUV and SLB are connected, allowing their outer membranes to merge. This connection facilitates the exchange of lipids between the two lipid structures through lateral diffusion, where lipid molecules move sideways within the tied monolayers. The hemifusion enables the exchange of lipid components from both membranes, creating asymmetric vesicles. The asymmetric vesicles are then detached from the SLB. Illustration adapted from [29].

Hemifusion is a different technique for engineering asymmetric membranes [18,30,98]. This alternative method involves the connection of giant unilamellar vesicles (GUVs) and a supported lipid bilayer (SLB) through their outer leaflets, as shown in Figure 10. The GUVs and the SLB are prepared with compositions meant for the inner and outer leaflets, respectively. Fusogenic agents, such as calcium ions, can induce hemifusion [99,100,101]. In the process of hemifusion, lipid exchange occurs exclusively in the connected outer leaflets through lateral diffusion. This efficient exchange allows lipids from the supported lipid bilayer (SLB) to replace the lipids on the outer leaflet of giant unilamellar vesicles (GUVs). The SLB, acting as a substantial reservoir of lipids, plays a crucial role in this highly efficient process. Different fluorescent probes label the GUVs and the SLB, aiding in the identification of lipid exchange. Thus, after hemifusion and lipid exchange, the newly formed asymmetric GUV (aGUV) comprises the two fluorophores originating in the GUVs or the SLB. Quantifying both fluorescence signals allows us to determine the percentage of lipids exchanged for individual vesicles. This powerful tool allows in-depth exploration of phase diagrams in asymmetric bilayers, offering a distinct advantage with this method.

Enoki and Feigenson (2019) conducted experiments to characterize hemifusion and prove that lipid transfer occurs only through the connected outer leaflets. The chelating of calcium ions after lipid exchange prevents complete bilayer fusion. The researchers have detailed the utilization of shear force to detach the aGUVs from the SLB [18].

We observe that the model indicates a composition that forms Ld and Lo phases in the inner leaflet of the GUV. This composition serves as a mimetic representation of the exoplasmic leaflet of the plasma membrane. Due to the GUVs’ minimal curvature effect, we can construct this model in the reverse order. It is essential that the experiments are conducted as shown to achieve a more controlled composition on the outer leaflet.

### 4.2. Electron Density Modeling for Asymmetric Bilayers

We can consider a straightforward model highlighting the differences between the lipid compositions modeling the plasma membrane leaflets. In this example, our asymmetric membrane has one leaflet with a composition that forms the coexistence of Ld and Lo phases, and another leaflet comprises a lipid composition that forms a single Ld phase. 

In order to enhance our understanding of the differences between leaflets, we evaluated the electron density profiles from leaflets containing different lipid compositions. Here, we simulate electron density profiles of symmetric bilayers to model (1) the Ld phase (leaflet Ld + Lo), (2) the Lo phase (leaflet Ld + Lo), and (3) the Ld phase (leaflet single phase) to build an asymmetric bilayer. Note that in our model of the asymmetric bilayer, at the bilayer midplane, there will be the contact of the compositions described by 1 and 3 and 2 and 3.

**Figure 11:**
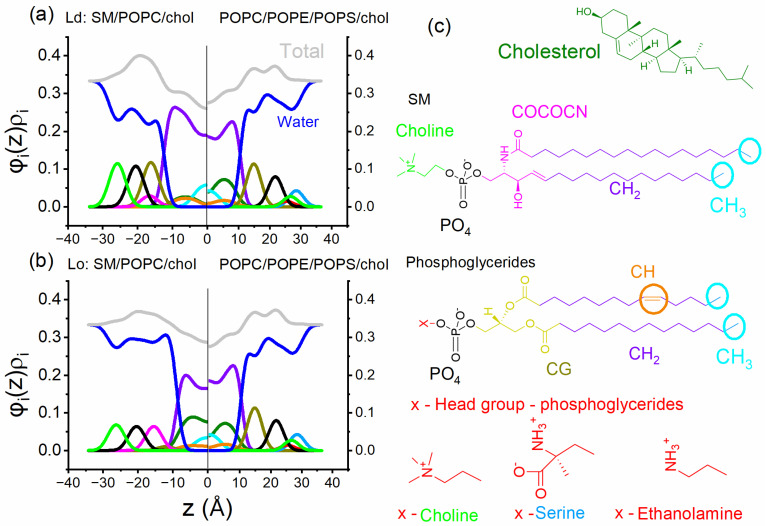
Electron density profiles calculated for the models (**a**) a membrane composed of SM/POPC/Chol = 0.22/0.71/0.07, which forms an Ld phase (left), and a membrane composed of POPE/POPS/POPC/Chol = 0.25/0.28/0.25/0.22 (right); (**b**) a membrane composed of SM/POPC/Chol = 0.62/0.08/0.3, which forms a Lo phase (left), and a membrane composed of POPC/POPS/POPE/Chol = 0.25/0.28/0.25/0.22 (right). The simulations are performed in symmetric membranes (see main text). Contraposing leaflets of different compositions assemble the asymmetric bilayer. (**c**) Illustration of the chemical group represented in the graphs (**a**,**b**). In the plots, each line represents the chemical group of the respective color. For example, dark green represents cholesterol.

In Figure 11, the Lo and Ld compositions have the lipid molar fractions SM/POPC/chol = 0.22/0.71/0.07 (a, Ld phase) and SM/POPC/chol = 0.62/0.08/0.3 (b, Lo phase). These compositions are obtained from the phase diagram and represent the endpoint of the bottom tieline, as shown in Figure 2c. In addition, in both panels (a and b), the model of the cytoplasmic leaflet comprises POPC/POPS/POPE/chol = 0.25/0.28/0.25/0.22. We note that we performed these calculations in symmetric bilayers, and here, we contrapose them together for comparison, though it may represent an asymmetric bilayer without any coupling.

Figure 11 shows the electron density profile ρi of each lipid chemical group weighted by its volume fraction φi within the lipid bilayer. Here, the parameter z represents a distance in angstroms (Å) perpendicular to the bilayer plane, with z = 0 set at the midplane of the bilayer. The total electron density profiles are represented by gray lines in Figure 11. The simulated distribution of the chemical groups in the bilayer was performed through the modified scattering density profile (MSDP) model [102], using GENFIT software [103]. For further details, the reader must refer to references [102,104]; the simulations are briefly described in the Supplementary Material.

Interestingly, the asymmetric electron density profile may create distinct lateral pressure between leaflets, affecting the partition of transmembrane peptides and proteins. Thus, Figure 11 emphasizes the potential impact of distinct lipid compositions on electron density profiles and the resulting physical properties. However, it prompts the question of whether asymmetric leaflets retain their intrinsic characteristics despite significant differences in their properties, such as order and lipid packing.

### 4.3. Experimental Phase Diagram of Asymmetric Lipid Bilayers: Domains and Coupling

Different experiments performed by different research groups on asymmetric bilayers have disclosed the appearance of induced domains on a leaflet expected to be uniform. First, the observations were made on asymmetric bilayers assembled on a support [14,15] and in (Montal–Mueller) membranes [27], as well as asymmetric vesicles [16,17,18,25,26,59,105,106]. Moreover, several experiments showed that asymmetric membranes prepared using cyclodextrin form induced domains but could also result in the vanishing of the phase separation [23,30,107], as discussed below.

Enoki and Feigenson (2019) and Enoki et al. (2021) report a particular example of induced domains [18,59]. In these experiments, the authors assembled asymmetric bilayers with one leaflet with a lipid composition of DSPC/DOPC/chol = 0.39/0.39/0.22, which formed Ld and Lo domains. In contrast, the second leaflet was enriched with DOPC/chol = 0.8/0.2, which displayed a fluid phase in the case of the symmetric membrane (Figure 3a). A surprising result in these asymmetric bilayers revealed that the DOPC/chol-rich leaflet, which was expected to be uniform, showed ordered domains.

**Figure 12:**
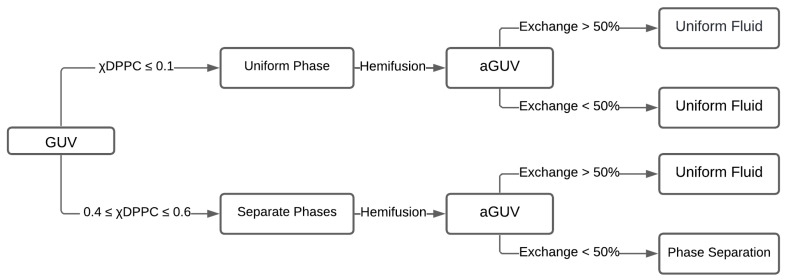
Phase behavior of asymmetric DPPC/DOPC giant vesicles. The diagram illustrates the preparation of the asymmetric GUV (aGUV) from the symmetric GUV (sGUV). The upper route in the diagram corresponds to sGUVs prepared with a concentration of DPPC up to 10%. The lower route corresponds to sGUVs prepared with a concentration of DPPC above 40%. The third column in the diagram separates aGUVs, which have obtained a percentage of lipid exchange of DPPC by DOPC below or above 50%. The last column indicates the phase presented by the corresponding aGUV.

A different effect, previously observed by different research groups, occurs when a phase separate and a fluid form an asymmetric membrane, and the fluid leaflet dictates the phase behavior of the entire bilayer. In particular, Enoki and Heberle (2023) reported a partial phase diagram from asymmetric DPPC/DOPC bilayers by contraposing theory and experiments [30]. The saturated lipid DPPC is a gel-forming lipid at room temperature, while pure DOPC bilayers display a disordered phase at ambient temperature. The asymmetry of the system results from different relative concentrations on the two leaflets. Figure 12 summarizes the phase behavior of the corresponding symmetric and asymmetric bilayers.

A necessary first step is the investigation of the phase behavior of symmetric membranes composed of DPPC/DOPC. A low fraction of the saturated lipid (DPPC ≤ 0.1) in symmetric bilayers yields a uniform, disordered phase. As the molar fraction of DPPC increases (0.2 < DPPC < 0.8), phase separation into gel + fluid phases sets in. Higher fractions of the saturated lipid (DPPC ≥ 0.9) result in faceted GUVs with a single uniform gel phase.

The second step is the preparation of aGUVs, engineered through the hemifusion technique. Briefly, the symmetric DPPC/DOPC GUVs were hemifused with a DOPC SLB, thus favoring the diffusion of DOPC into the GUV’s outer leaflet. The inner leaflet maintains the initial lipid composition of the symmetric GUVs.

The GUVs and the SLB were previously doped with different fluorescent probes to control the asymmetry: DiD fluorophore (red) labels the symmetric GUV, whereas TFPC (green) labels the DOPC SLB. After hemifusion, the green dyes provide information about the behavior of the outer leaflet of the aGUVs. The percentage of lipid exchange can be calculated by examining the signal of both dyes on individual aGUVs.

The fluorescence signals used to control asymmetry also report the presence or absence of phase separation. The authors thus establish the phase behavior of the asymmetric GUVs (aGUVs) and compare it with that of the symmetric GUVs.

The asymmetric vesicles followed the symmetric membrane behavior for a low fraction of the saturated lipid (molar fraction DPPC ≤ 0.1) in the symmetric GUV. They exhibited a uniform fluid phase, independently of the degree of outer leaflet exchange. Thus, for a low DPPC fraction in the original symmetric vesicle, aGUVs present the exact morphology of the symmetric vesicles.

When the saturated lipid is present in intermediate fractions (0.4 ≤ DPPC ≤ 0.6) in the inner leaflet and less than 50% lipid exchange occurs for the outer leaflet, aGUVs exhibit phase separation in both leaflets; however, if the exchange in the outer leaflet is over 50%, aGUVs turn into uniform vesicles under observation in an ordinary microscope. Surprisingly, the phase domains previously present in the inner leaflet vanish or have reduced domain sizes below 200 nm, the microscope resolution.

These experimental results suggest an interpretation in terms of interaction between leaflets: an energetic penalty in the midplane between ordered and disordered phases in opposing leaflets, which could compete with the interactions driving phase separation.

The presence of induced domains, or the second case in which the asymmetric membranes show a single phase, has been described in terms of an “inter-leaflet coupling” [108,109]. The origin of this possible coupling is under discussion in different publications [29,110,111,112,113].

### 4.4. Theoretical Minimal Models

Theoretical models play a crucial role in elucidating lipid interactions, and our comprehension of this complex system has significantly advanced over the last 50 years [114]. For example, intra-leaflet lipid–lipid interactions can explain the main gel–fluid transition for single lipid membranes or the ripple phase [115,116,117,118]. Furthermore, the fascinating dynamics of intra-leaflet interactions can be beautifully captured through pairwise interactions, offering us a powerful way to model line tension [119].

The new experimental results on asymmetric membranes have triggered the interest of the membrane community with a new question: What is the rationale of the “inter-leaflet coupling”? Several hypotheses are under investigation: interdigitation, cholesterol distribution between leaflets, and inter-leaflet interactions, among others.

**Figure 13:**
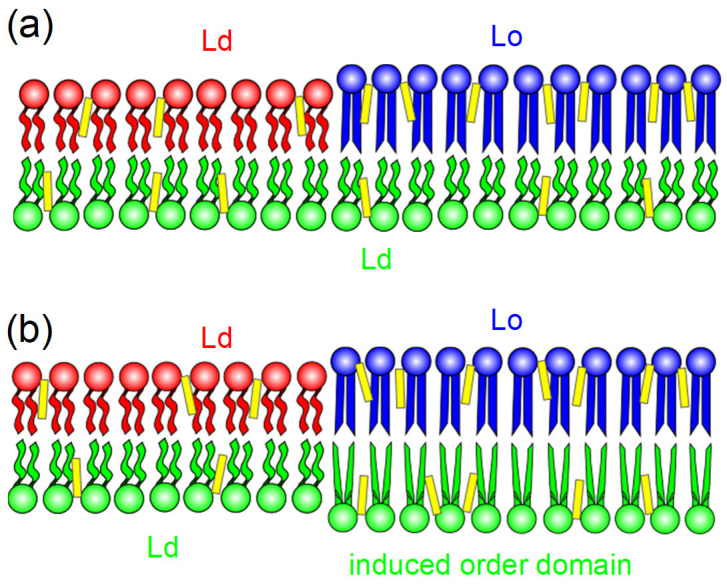
Representation of interacting leaflets in asymmetric vesicles. (**a**) Uncoupled leaflets: the exoplasmic leaflet (disordered-chain green lipids) displays an Ld phase, while the cytoplasmic leaflet displays the coexistence of an Ld (disordered-chain red lipids) and Lo phase (straight-chain blue lipids). (**b**) Coupled leaflets: induced ordered domain in the exoplasmic leaflet (ordered green lipids are represented by straight chains) associated with the Lo domain region of the cytoplasmic leaflet. Note that cholesterol (represented in yellow) prefers the region of ordered lipids.

In this mini-review, we focus on the hypothesis that there is a coupling interaction between leaflets of asymmetric bilayers since the domains that appear on the leaflet composed of low Tm lipids frustrate the previous idea of negligible coupling between the apposed monolayers. In this case, a plausible explanation could be an interaction between the leaflets responsible for the formation of induced ordered domains within the leaflet of the fluid (low Tm) lipid composition. Figure 13a,b illustrate this hypothesis. In Figure 13a, each leaflet of the asymmetric bilayer displays an independent behavior, suggested by the phase diagrams of the two corresponding symmetric membranes. In contrast, Figure 13b illustrates the possible phase behavior of the two leaflets in accordance with the observed behavior in the experiments displaying induced ordered domains.

The likely cause of the formation of the induced ordered domain is an energetic penalty between Lo/Ld contacts within the intermediate plane of the bilayer. The energy penalty would result in the lipids rearranging, reducing unfavorable contacts between ordered and disordered chains, thereby decreasing interfacial tension. Enoki and Heberle (2023) have compared their experimental results for the phase diagram of the asymmetric lipid bilayer with predictions of a simple mean-field model proposed by Wagner et al. (2007) [108]. It is important to note that they utilized a binary mixture that coexists in gel and fluid phases. While comparison with Ld and Lo coexistence systems is possible, it must be conducted with caution.

To explore how different phases can affect each other on opposing leaflets, Wagner et al. considered a regular solution model, referred to as the Bragg–Williams or random mixing approximation [120]. This model includes an interaction parameter that penalizes different phases on opposing leaflets. In this model, the free energy per lipid of a single leaflet that comprises a binary mixture can be written as a function of its composition, as shown in Equation (5):

(5) $$f_{BW}(\phi )=\phi ln\phi +(1-\phi )\ln\left(1-\phi \right)+Χ\phi (1-\phi ).$$

where ϕ is the lipid composition, and all energies are expressed in units of k_B_T (Boltzmann’s constant × absolute temperature). The parameter X represents the effective strength of nearest-neighbor interactions. A more general approach considers each leaflet to have a particular X, as reported [109].

Wagner et al. (2007) suggest writing the local free energy of an asymmetric bilayer according to Equation (6), where Λ (Λ > 0) denotes the coupling strength.

(6) $$f_{BW}(\phi ,\psi )=f_{BW}(\phi )+f_{BW}(\psi )+{\Lambda (\phi -\psi )}^{2},\;$$

where ϕ and ψ are local compositions on individual leaflets.

Accordingly, if the compositional difference is sufficiently small, any local compositional variations across the bilayer result in an additional energy cost that should be proportional to ${\left(\phi -\psi \right)}^{2}$. Then, to characterize the phase behavior, the authors calculate the spinodal lines by taking the second derivative of the free energy to zero. For a given value of X, it is possible to discuss how the interplane interaction Λ affects these spinodal lines, limiting how stable a system can be against phase separation when it experiences small fluctuations. In the model, the interplane Λ interaction, representing midplane interfacial tension, competes with an in-plane lipid–lipid interaction X. The theoretical model roughly reproduces the experimental phase diagram for intermediate values of the interplane Λ interaction (0.35 < Λ < 0.55) parameter at fixed X = 2.25. Λ and X are dimensionless model parameters. However, Λ is proportional to the midplane surface tension parameter, γ, proposed by Wagner et al. (2007) [108], and our model parameters produce results for the midplane surface tension value that are compatible with the values reported in molecular dynamics simulation studies [121].

**Figure 14:**
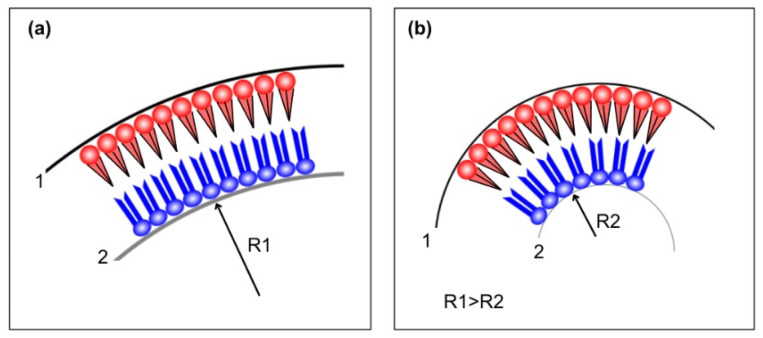
Illustration depicting the interplay between optimal curvature and ideal tension for each leaflet. (**a**) Leaflet 1 (in red) displays a bending torque, which tends to increase curvature due to the conic-like lipid form, while leaflet 2 (in blue) remains tensionless. (**b**) Leaflet 1 achieves its “natural” curvature, while leaflet 2 gains negative tension.

A different approach to inter-leaflet interaction relies on the competition between elastic energies. Lipids exhibit different intrinsic curvature depending on headgroup tail sizes and on-chain rigidity [122]. The presence of curvature involves elastic energy, which can be described as bending energy dependent on the bending modulus of the specific molecular structure. For symmetric vesicles, the bending rigidity is the same on the inner and outer leaflets, and the system relaxes to zero area tension on each leaflet. As for the asymmetric case, the situation is more complex. The difference in composition between the two leaflets is the source of additional area stress. In other words, different lipid geometries impair an ideal area relaxation for the two leaflets [123,124]. However, a second factor is present: each leaflet presents its own rigidity and, therefore, a preferential curvature, while, at the same time, each leaflet displays its own area tension. The two effects may oppose each other (as represented in the cartoon, Figure 14), which can be described as competing terms in elastic energy.

Thus, bending stress remains if one considers a relaxed overall area tension. Otherwise, if bending stress is relaxed, area stress cannot be relaxed simultaneously. Hossein and Deserno (2020) have explored the two features and showed that even in the minimum energy situation, with zero overall stress, differential stress remains between the two leaflets [125].

Cholesterol may be unable to balance this stress if the cholesterol molecules prefer the leaflet modeling the exoplasmic leaflet due to its preference for saturated lipids. However, the distribution of cholesterol between leaflets is still controversial. Hossein and Deserno (2020) investigated the competition analytically and through coarse-grained models and have shown that the differential stress may contribute to an increased bending modulus and a transition to the gel phase of the compressed leaflet [125]. The investigation of the possibility is an open road to exciting research for membrane experts, either through *in vitro* experiments, theory, or computational experiments.

## 5. Summary

In this short review, we have shown the importance of constructing lipid phase diagrams to guide the study of the cell membranes of different compositions. Many techniques are available for identifying the coexistence of phases. An important question from the point of view of natural membranes is the establishment of the size of the ordered/disordered domains, which might be related to rafts seen in natural cell membranes.

Thus, as a first step in investigating lipid bilayers, experimentalists have studied different symmetric bilayers mimicking the cytoplasmic or exoplasmic leaflet of the plasma membrane. These studies allowed the construction of several ternary and quaternary phase diagrams and the discovery of nanoscopic domains.

As a second step, recent studies have approached binary lipid asymmetric systems. Hemifused asymmetric bilayers were shown to produce induced order (or induced disorder) domains, highlighting the necessity of considering inter-leaflet coupling in asymmetric bilayers. Initial modeling of this new phenomenon has been presented in terms of direct coupling due to inter-leaflet tension and elastic coupling.

In addition, the field is open to exciting new research in the laboratory, in modeling design, and using computers. Ternary asymmetric bilayers must be explored for phase and domain properties; the nature of coupling requires further testing, and the impact of asymmetry on transmembrane protein localization needs investigation; these are just some of the open questions in the new adventures for membrane scientists.

## Data Availability

No new data were created or analyzed in this study. Data sharing is not applicable to this article.

## Supplementary Materials

The following supporting information can be downloaded at: https://www.mdpi.com/article/10.3390/membranes15030079/s1.

## Abbreviations

AFM	Atomic Force Microscopy
aGUVs	Asymmetric Giant Unilamellar Vesicles
bSM	Brain Sphingomyelin
CDs	Cyclodextrins
Chol	Cholesterol
DLS	Dynamic Light Scattering
DHE	Dehydroergosterol
DOPC	1,2-Dioleoyl-sn-glycero-3-phosphocholine
DOPE	1,2-Dioleoyl-sn-glycero-3-phosphoethanolamine
DPPC	1,2-Dipalmitoyl-sn-glycero-3-phosphocholine
DSPC	1,2-Distearoyl-sn-glycero-3-phosphocholine
ESR	Electron Spin Resonance
FRET	Förster Resonance Energy Transfer
GUV	Giant Unilamellar Vesicle
Kp	Partition Coefficient
LUV	Large Unilamellar Vesicle
Ld	Liquid disordered phase
Lo	Liquid ordered phase
MSDP	Modified Scattering Density Profile
PC	Phosphatidylcholine
PE	Phosphatidylethanolamine
PEp	Phosphatidylethanolamine plasmalogen
PLD	Phospholipase D
PM	Plasma Membrane
POPG	1-Palmitoyl-2-Oleoyl-sn-glycero-3-[Phospho-rac-(1-glycerol)]
PS	Phosphatidylserine
SANS	Small-Angle Neutron Scattering
SAXS	Small-Angle X-ray Scattering
SLB	Supported lipid bilayer
SM	Sphingomyelin
SSPP	Steady-State Probe Partition
TFPC	1-palmitoyl-2-(dipyrrometheneboron difluoride)undecanoyl-sn-glycero-3-phosphocholine
