# Supplementary material for: The Importance of Bilayer Asymmetry in Biological Membranes: Insights from Model Membranes
Source: Membranes (Basel). 2025 Mar 3;15(3):79. doi: 10.3390/membranes15030079 (PMC11943618; doi:10.3390/membranes15030079)
Supplement: Supplementary file 1 [file membranes-15-00079-s001.zip › membranes-3369848-supplementary.pdf]

Supporting Information

Exploring the plasma membrane lipid interactions and properties in experimental models

Igor S. Oliveira †, Guilherme X. Pinheiro †, Maria Lauana B. Sa, Pedro Henrique L.O. Gurgel, Samuel U. Pizzol, Rosangela Itri, Vera B. Henriques and Thais A. Enoki.

Institute of Physics, University of Sao Paulo, Sao Paulo, Brazil

* Correspondence: enokita@if.usp.br

† The authors have contributed equally to this work

Simulations of electron density profiles

We used The Modified Scattering Density Profile model, as previously described by (1). We characterize the distribution of volume fractions for each i-chemical group, using a combination of two error functions according to the equation (S1) (except for CH2, along the z-axis, which is perpendicular to the plane of the bilayer)

$$E_2(z, z_i, w_i, \sigma_i) = \frac{1}{2}\left[erf\left(\frac{z - z_i + w_i}{2^{\frac{1}{2}}\sigma_i}\right) - erf\left(\frac{z - z_i - w_i}{2^{\frac{1}{2}}\sigma_i}\right)\right], \qquad \text{(S1)}$$

where z_i represents the location of the peak, $2w_i$ indicates the width of the peak, and σ_i refers to the standard deviation of the error function. In this case, we used a symmetrical bilayer.

Consequently, two peaks at symmetrical points $\pm z_i$ are characterized by (2)

$$E_{2S}(z, z_i, w_i, \sigma_i) = E_2(z, z_i, w_i, \sigma_i) + E_2(z, z_i, w_i, \sigma_i), \qquad \text{(S2)}$$

The volume fraction distribution of a particular i-group is given by equation (S4)

$$\varphi_i(z) = \frac{n_i v_i [\, E_2(z, z_i, w_i, \sigma_i) + E_2(z, z_i, w_i, \sigma_i) \,]}{2Aw_i}. \qquad \text{(S4)}$$

where v_i represents the molecular volume of the i-group, n_i is the number of i-groups in the lipid molecule. A is the area per molecule, and V is the total hydrophobic volume (2).

The scattering length densities (SLD) of the bilayer is defined in equation (S5)

$$\rho(z) = \sum_{i=1}^{N} \frac{\varphi_i\,(z) b_i}{v_i}. \qquad \text{(S5)}$$

where b_i is the scattering length of the group, representing the number of electrons carried on by the group multiplied by the classical radius of the electron (r_e = 0.28 · 10^{-12} cm).

References:

1. Rosa, R. De, F. Spinozzi, and R. Itri. 2018. Hydroperoxide and carboxyl groups preferential location in oxidized biomembranes experimentally determined by small angle X-ray scattering: Implications in membrane structure. Biochim. Biophys. Acta - Biomembr. 1860: 2299–2307.

2. Kučerka, N., J.F. Nagle, J.N. Sachs, S.E. Feller, J. Pencer, A. Jackson, and J. Katsaras. 2008. Lipid bilayer structure determined by the simultaneous analysis of neutron and X-ray scattering data. Biophys. J. 95: 2356–2367.
